# Editorial: Biosafety and Biosecurity Approaches to Counter SARS-CoV-2: From Detection to Best Practices and Risk Assessment Volume 2

**DOI:** 10.3389/fbioe.2022.1118544

**Published:** 2023-01-06

**Authors:** Segaran P. Pillai, Stephen A. Morse

**Affiliations:** ^1^ Office of the Commissioner, United States Food and Drug Administration, Department of Health and Human Services, Silver Spring, MD, United States; ^2^ Centers for Disease Control and Prevention (Retired), Department of Health and Human Services, Atlanta, GA, United States

**Keywords:** biosafety & biosecurity, SARS—CoV—2, detection and diagnostics, PPE (personal protection equipment), best praclices

Severe acute respiratory syndrome coronavirus 2 (SARS-CoV2) is the causative agent of Coronavirus Disease-19, commonly referred to as COVID-19. SARS-CoV- 2 was discovered in 2019 and is currently responsible for a global pandemic that has resulted in more than 642.7 million cases and 6.625 million deaths worldwide as of 19 November 2022. However, there have been a large number of asymptomatic cases that have gone unreported, which likely results in an overestimation of the case fatality rate of 1.03%. It is also important to note that many deaths due to SARS-CoV-2 have gone unreported.

Since the pandemic associated with SARS-CoV-2 began, countless laboratories around the world have switched their research priorities to actively work on this virus. Much of the research to date has focused on the origin of the virus, its pathogenicity, development of vaccines and medical countermeasures such as anti-virals, immune globulins, rapid diagnostics and detection technologies, surveillance efforts and the monitoring of genetic changes and the emergence of new variants. The latter includes Alpha (B.1.1.7 and Q lineages), Beta (B.1.351 and descendent lineages), Gamma (P.1 and descendent lineages), Delta (B.1.617.2 and AY lineages), Epsilon (B.1.427 and B.1.429), Eta (B.1.525), lota (B.1.526), Kappa (B.1.617.1), 1.617.3, Mu (B.1.621 and B.1.621.1), Zeta (P.2) and B.1.617.3 variants which are being monitored as well as variants of concern which includes Omicron (B.1.1.529, BA.1, BA.1.1, BA.2, BA.3, BA.4, and BA.5 lineages) (CDC, 2019).

The current observation of the virus’s ability to mutate and adapt begs the need for enhanced biosafety and biosecurity measures to ensure that the laboratory work being performed does not contribute to laboratory-acquired infections (LAI) or give rise to new variant(s) that are accidentally introduced into the community through a LAI. To ensure this, there is a critical need for laboratories conducting such work to perform risk assessments and implement appropriate risk mitigation strategies along with safe and best laboratory practices leveraging engineering and administrative controls, personal protective equipment, etc. To this end, Frontier’s developed a Research Topic entitled “Biosafety and Biosecurity Approaches to Counter SARS-CoV-2: From Detection to Best Practices and Risk Assessments Volume 2” to enhance scientific communications. Volume 1 received 34 submissions from 14 countries reflecting the global nature of the pandemic and the urgency that it spawned. Volume 2 had far fewer submissions, which may have reflected the subsequent development, approval and use of effective vaccines and other products, and the alleviation of some supply chain Research Topic.

In this Research Topic (Buhr et al., 2022), shared critical information about 14 ultraviolet decontamination technologies and their efficacy against a SARS-CoV-2 surrogate that was dried onto different surfaces for laboratory and field testing. Their studies showed that ultraviolet (UV) decontamination technologies exhibited a wide range of variability with respect to dosage, efficacy, hazards and UV output over time and that each UV device needed independent technical measurements and assessment for product development prior to and during use.

The role of Dual Use Research of Concern, sometimes referred to as Gain of Function (GOF) and Enhanced Potential Pandemic Pathogens (EPPP) policies have been debated for quite some time and are being revaluated due to the current COVID-19 pandemic (Shinomiya et al., 2022). discussed the history of GOF research and its significance considering the current COVID-19 pandemic and the directions that should be taken in the future.

Rapid diagnostics and self-isolation are a critical element for preventing disease transmission. The need for an inexpensive, rapid, readily assessable, and easy-to-use test would be of tremendous benefit for disease mitigation and controlling spread (Zhang et al., 2022). discussed the essential features of SARS-CoV-2, compared existing detection methods and focused on the principles, merits and limitations of Lateral Flow Assays (LFAs) that detect viral nucleic acids, antigens and/or corresponding antibodies. They also provided a comprehensive assessment of the LFA technology and insights into preventing and curbing the COVID-19 pandemic.

One of the challenges the world faced during the pandemic was the demand for respiratory protection. Scientist from around the world have worked diligently to overcome shortfalls by decontaminating and reusing masks. (Obrová et al., 2022), compared the different decontamination strategies (e.g., non-thermal plasma [NTP]) to other methods such as dry heat and ultraviolet light) for decontaminating personal protective equipment such as respirators, which are designed to provide high efficiency filtration. They showed that NTP treatment completely inactivated SARS-CoV-2 and other respiratory pathogens such as Influenza A, Rhinovirus and Adenovirus. They also showed that unlike methods such as autoclaving, NTP did not influence the filtering efficiency nor the microstructure of the filter membrane and demonstrated that NTP is a powerful technique for decontamination of sensitive equipment such as respirators and protective filters for reuse.

Biosafety and biosecurity serve as the cornerstone for laboratory containment of highly transmissible and pathogenic microorganism to protect public health by preventing the next outbreak, epidemic or pandemic through unintentional or accidental introduction.

Although we have learned a lot over the past several years about SARS-CoV-2 and its associated disease, COVID-19, there still remains a need for better and more efficacious medical countermeasures, supportive therapies, diagnostics and detection technologies, decontamination technologies, surveillance methods, and disease containment measures if we’re to eradicate or efficiently manage this disease.

Finally, we would like to acknowledge the contributions and thank the many scientists around the world for their work in enhancing our knowledge about this disease and for their dedication to public health.
